# Physiological Responses of Two Epiphytic Bryophytes to Nitrogen, Phosphorus and Sulfur Addition in a Subtropical Montane Cloud Forest

**DOI:** 10.1371/journal.pone.0161492

**Published:** 2016-08-25

**Authors:** Xi Chen, Wen-yao Liu, Liang Song, Su Li, Yi Wu, Xian-meng Shi, Jun-biao Huang, Chuan-sheng Wu

**Affiliations:** 1 Key Laboratory of Tropical Forest Ecology, Xishuangbanna Tropical Botanical Garden, Chinese Academy of Sciences, Kunming, China; 2 University of Chinese Academy of Sciences, Beijing, China; Tennessee State University, UNITED STATES

## Abstract

Atmospheric depositions pose significant threats to biodiversity and ecosystem function. However, the underlying physiological mechanisms are not well understood, and few studies have considered the combined effects and interactions of multiple pollutants. This *in situ* study explored the physiological responses of two epiphytic bryophytes to combined addition of nitrogen, phosphorus and sulfur. We investigated the electrical conductivity (EC), total chlorophyll concentration (Chl), nutrient stoichiometry and chlorophyll fluorescence signals in a subtropical montane cloud forest in south-west China. The results showed that enhanced fertilizer additions imposed detrimental effects on bryophytes, and the combined enrichment of simulated fertilization exerted limited synergistic effects in their natural environments. On the whole, EC, Chl, the effective quantum yield of photosystem II (Φ_PSII_) and photochemical quenching (qP) were the more reliable indicators of increased artificial fertilization. However, conclusions on nutrient stoichiometry should be drawn cautiously concerning the saturation uptake and nutrient interactions in bryophytes. Finally, we discuss the limitations of prevailing fertilization experiments and emphasize the importance of long-term data available for future investigations.

## Introduction

On a global scale, the fluxes of nitrogen (N), phosphorus (P) and sulfur (S) have increased by 108%, 400% and 113%, respectively, due to human intervention [1]. Anthropogenic N pollutions, originating mainly from intensive agriculture activities and fossil fuel combustion processes [2], pose significant threats to biodiversity and ecosystem function [3]. As a robust bioindicator, bryophytes are particularly susceptible to atmospheric pollutions due to their unique morphological features and specific characteristics of nutrients uptake [4]. However, most research to date has focused on the response of vascular plants, and bryophytes have received far less attention. Moreover, much of our knowledge on the effects of N deposition on bryophytes is derived predominantly from *Sphagnum* species in peatlands, where nutrient limitation is a peculiar biogeochemical feature [5]. By contrast, montane areas are potentially at a greater risk of severe pollution events than surrounding lowlands [3] because of increased precipitation and higher pollutant concentration at higher altitudes [6]. Therefore, the increase in both the concentration and total dose with altitude may exert profound impacts on bryophytes in upland ecosystems.

If humans continue to preferentially increase N deposition, some ecosystems may shift from N-limitation to P-limitation [7, 8]. Meanwhile, anthropogenic activities simultaneously cause severe P pollution, and long-range aerosol transport is an important contributor to determine atmospheric P concentration at the deposited site [9]. For example, coal soot derived from China acts as a crucial source of atmospheric P deposition in Ashiu, central Japan [10]. In addition, eastern Asia has become the third largest acid rain region due to acid deposition after Europe and North America. Chinese acid rain is characterized by high concentrations of SO_4_^2−^ and NH_4_^+^ and a low concentration of NO_3_^−^, which makes it fall into a typical sulfate type [11]. S pollution is mainly influenced by local emissions, while long-range transport also plays an important role in determining local concentration of S deposition. In some heavily exposed areas of China, total S deposition has been estimated to be about 100 kg ha^−1^ yr^−1^ [12].

Numerous N addition experiments have demonstrated profound ecological effects on a variety of habitats. For example, cumulative N deposition leads to a decline in species richness [13], abundance [14, 15] and community uniqueness [16]. However, the underlying physiological mechanisms still remain unclear. Physiological signals are expected to provide in-depth information on species-specific responses, because they are more responsive and readily detectable compared with measurements of growth and abundance, which are usually difficult to quantify with high precision in bryophytes [17]. Much of our current knowledge on the impacts of N deposition comes from field manipulative experiments. Limited fertilization experiments have suggested that elevated N input may cause imbalance in nutrient stoichiometry [5, 8]. These experiments are invaluable in determining the impacts of N deposition on community composition and in elucidating the potential mechanisms, but they usually use pulse additions over a relative short timescale, in an attempt to simulate the high cumulative dose that results from chronic atmospheric deposition [18]. Hence, the results of short-term studies must be interpreted with caution. In fact, they sometimes yield contradictory results, which may be attributed to the fact that levels of N deposition at the study sites, as well as the amounts of applied N, varied considerably.

The particular effects of an individual nutrient addition have been studied extensively, while few studies have considered their combined effects and interactions [19]. Given that these variables change simultaneously, in depth analyses on their combined effects are necessary in order to understand and predict future effects of global change on montane forest ecosystems. For instance, previous research showed that the responses of bryophytes to N addition depend on the co-limited levels of P and potassium (K) [5, 20], but these outcomes vary significantly among studies. Limpens and others [21] concluded from a four-year fertilization experiment that P addition alleviates the negative effect on *Sphagnum* by enhancing the assimilating capability of increased N. Similarly, Carfrae and others [22] found that, at least in the short-term, additions of P and K to N-polluted bogs counteract the detrimental impacts of enhanced N on *Sphagnum capillifolium*. In contrast, other investigators revealed that the increasing availability of P and K with N fails to alleviate the physiological stress imposed by excessive N [23], or even exacerbates the negative impact on sustainability of peatland [20, 24].

There are many hypotheses about which nutrient may affect the response of epiphytic bryophytes to N enrichment, but the relative importance among these covariant nutrients remains poorly understood, as does whether their combined effects can be distinguished from an individual fertilization experiment [25]. For example, several studies have provided valuable insights into the negative effects of increased N and P input on bryophyte production and species richness [26, 27], while it remains unclear which fertilizer enrichment is the dominant driver of species loss from montane forests ecosystems. The main reason is that very few studies have directly addressed the relative impact of N vs P enrichment by measuring both N and P availability. In some cases, multivariate statistics can tease apart the relative effects of N from other factors. But under most conditions, it is difficult to distinctly separate the correlated stressors from each other.

Given the status of local soil as P-deficient [28], we hypothesize that N fertilization has adverse impacts on physiological performance of epiphytic bryophytes, whereas the combined fertilization with P and S would mitigate the physiological stresses induced by excessive N. We investigated the physiological responses of two ecologically related but taxonomically distinct bryophytes to nutrient addition with the purpose of addressing two main issues: (1) whether the combined additions of P and S with N could mitigate the adverse physiological responses in two epiphytic bryophytes; and (2) which physiological parameters are the more reliable indicators of increased artificial fertilization.

## Materials and Methods

### Study site and experimental design

The manipulative experiment was carried out in a subtropical montane cloud forest in the Ailao Mountains National Nature Reserve (23° 36′–24° 56′ N 100° 44′–101° 30′ E). Montane cloud forest accounts for nearly 80% of the total area of the Reserve. Co-dominant tree species include *Lithocarpus xylocarpus* (Kurz) Markgr., *Lithocarpus hancei* (Benth.) Rehder, *Castanopsis wattii* (King ex Hook. f.) A. Camus, *Schima noronhae* Reinw. ex Blume and *Stewartia pteropetiolata* W. C. Cheng. The high annual precipitation and relative humidity allow the forest to harbor abundant epiphytes. Meteorological observations show that mean annual precipitation is approximately 1947 mm, with 85% falling in the rainy season (May–October). The mean annual relative humidity is 85% and mean annual temperature is 11.3°C.

Three plots were established in the study region. In each plot, two arboreal bryophyte species, *Homaliodendron flabellatum* (Sm.) Fleisch. and *Plagiochila assamica* Steph., were chosen as target species because they are abundant and widespread in the study region. No specific permits were required for the described field study, as some plots were designedly separated for scientific research and no endangered or protected species were involved in the study. Given that fertilization experiments are labor-intensive, expensive and usually limited by spatial scale, they probably misestimate the consequences of elevated atmospheric deposition [29]. As an alternative labor-saving and cost-effective approach, an orthogonal design *L*_27_(3^13^) (S1 Table, three factors with three levels each) with three replications (*n* = 3) was employed to investigate the main effects and interaction effects on the physiological responses of two epiphytic bryophytes. In total, 81 field quadrats of 3.5×3.5 m were established randomly. These experimental quadrats, separated by at least a 5.0 m buffer zone, were located in the same region at similar altitudes and slopes.

Background wet deposition in the area is estimated at 10.5 kg N ha^−1^ yr^−1^, 1.35 kg P ha^−1^ yr^−1^ and 2.83 kg S ha^−1^ yr^−1^ [30]. The treatments commenced in April 2012 and lasted for 30 months. Field quadrats were sprayed with NH_4_NO_3_, NaH_2_PO_4_ and Na_2_SO_4_ solutions at three levels (i.e. at doses equivalent to 10, 20 or 30 kg N ha^−1^ yr^−1^, 3, 6 or 12 kg P ha^−1^ yr^−1^ and 6, 12 or 24 kg S ha^−1^ yr^−1^, respectively, with ambient nutrient inputs not included). Fertilizer solutions were applied to the quadrats as a fine mist using a spray bottle twice per month. At each application, the chemicals for each quadrat were dissolved in 1.5 L deionized water, which ensured that the bryophytes were fully hydrated. The pH of all fertilizer solutions was adjusted to pH 5.0.

### Electrical conductivity

The sampling program was undertaken over two consecutive weeks in October 2014. Ten days after the last N spraying event, samples of approximately 350 mg were taken from each treatment and immersed for 1 h in 100 ml deionized water, stirred with a glass rod at an interval of 5 minutes. The electrical conductivity (EC) was measured (*S*_1_, μS cm^–1^) by a conductivity meter (DDSJ-308A, Shanghai Precision & Scientific Instrument Co. Ltd, China). Capitula were then boiled for 10 mins to cause total rupture of cell membranes, and conductivity was measured repeatedly (*S*_2_, μS cm^–1^) after samples were cooled to room temperature. Relative conductivity was expressed as the percentage ratio between the two conductivities (EC = *S*_1_/*S*_2_×100%).

### Chlorophyll concentrations

Total chlorophyll concentration (Chl) was extracted in 95% ethanol by grinding tissues with a mortar and pestle, and measured by a spectrophotometer (UV-B 2501, Shimadzu, Japan). The Chl concentration was calculated on an air-dried-mass basis according to the extinction coefficients of Arnon [31].

### Chemical analyses

Samples were oven-dried at 65°C for 48 h to constant mass and only the apical sections were used for chemical analyses. Total N concentrations were determined using a CN analyzer (Vario MAX CN, Elementar Analysensysteme GmbH, Germany); total P and total S were measured by an inductively coupled plasma atomic-emission spectrometer (iCAP6300, Thermo Fisher Scientific Inc., USA).

### Chlorophyll *a* fluorescence measurements

Chlorophyll *a* fluorescence signals were measured with a PAM fluorometer (FMS2, Hansatech Instruments Ltd., UK) on two capitula. The capitula were saturated with deionized water and dark-adapted for 23 mins prior to the measurements. Samples were exposed to a weak modulated beam to assess the initial minimal fluorescence efficiency in the dark-adapted state (F_0_). A saturation pulse of approximately 5500 μmol m^−2^ s^−1^ for 0.7 s was then given to assess the maximal photochemical efficiency when photosystem II (PSII) centers are closed (F_m_). Opening the actinic illumination (approx. 110 μmol m^−2^ s^−1^) and saturating illuminations, the minimal/maximal Chl fluorescence efficiency (F_0_′/F_m_′) as well as the steady-state Chl fluorescence efficiency in the light–adapted state (F_s_) were measured, respectively. Using these parameters, the following ratios were calculated: maximal photochemical efficiency of PSII in the dark-adapted state: F_v_/F_m_ = (F_m_−F_0_)/F_m_ (F_v_, variable fluorescence yield); actual photochemical efficiency of PSII in the light-adapted state: Φ_PSII_ = (F_m_′−F_s_)/F_m_′; photochemical quenching: qP = (F_m_′−F_s_)/(F_m_′−F_0_); non-photochemical quenching: NPQ = (F_m_−F_m_′)/F_m_′.

### Data analyses

All data were submitted to normality and homogeneity tests before further statistical analysis. General linear model was used to compare the physiological parameters among different treatments, and multiple comparisons were conducted with least significant difference (*LSD*) or Games-Howell post hoc tests at a significance level of *P*≤0.05. All statistical analyses were performed with SPSS17.0 (SPSS Inc., USA).

## Results

### Electrical conductivity

**Fig 1:**
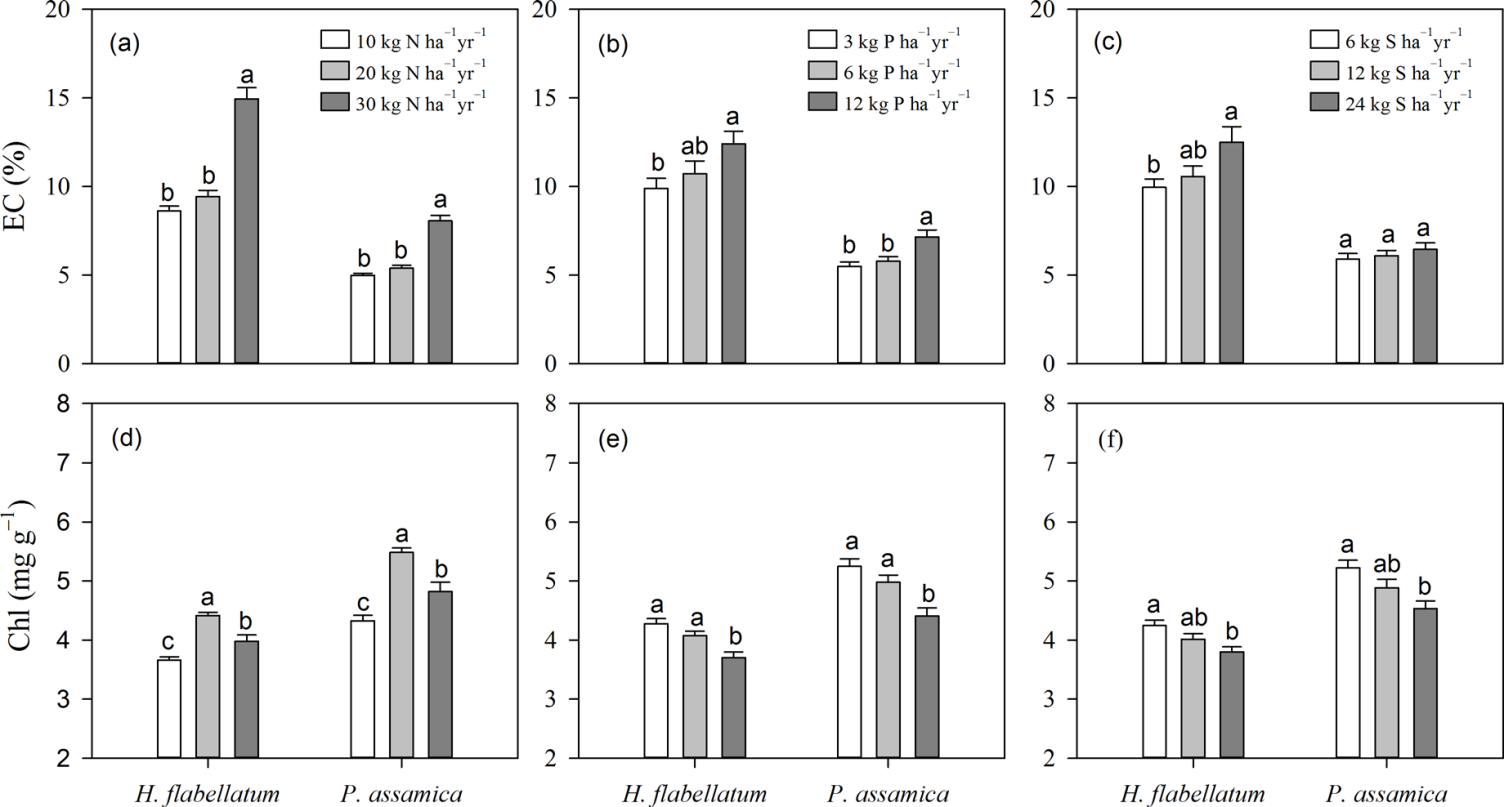
Multiple comparisons of different fertilizers and application levels on EC and Chl of *H*. *flabellatum* and *P*. *assamica* (means±se, *n* = 3). Three application levels are equivalent to 10, 20 and 30 kg N ha^−1^ yr^−1^, 3, 6 and 12 kg P ha^−1^ yr^−1^ and 6, 12 and 24 kg S ha^−1^ yr^−1^, respectively. Letters above columns indicate significant differences (*P*≤0.05).

Artificial fertilizer application for 2.5 years interfered with the physiological performance of the two target bryophytes (S2 Table). As hypothesized, fertilizer addition caused loss of membrane integrity and increased solute leakage in the two bryophytes (Fig 1A–1C). Overall, a dramatic increase in EC was observed with increased chemical fertilization. However, the response of the two bryophytes to fertilizer addition was species specific. Contrary to our expectation, application of additional P to *H*. *flabellatum* did not alleviate the detrimental impact of high-level N addition on EC, as a weak interaction between N and P was observed (*N*×*P*, Table 1; *P*>0.05). While S fertilization significantly alleviated the negative impact of high-level N on EC (*N*×*S*, Table 1; *P*<0.001). Moreover, the interaction between P and S was not so distinct (*P*×*S*, Table 1; *P*>0.05). In the case of *P*. *assamica*, the interaction between N and P was significant (Table 1; *P*<0.05), while S fertilization did not alleviate the detrimental impact of high-level N and P on EC (Table 1; *P*>0.05).

**Table 1:** Results of ANOVA for three factors and interactions affecting physiological responses of *H*. *flabellatum* and *P*. *assamica* by an orthogonal design *L*_27_(3^13^) (*n* = 3; *df*_t_ = 26, *df*_e_ = 54).

Sources	EC		Chl		[N]		[P]		[S]		N/P		F_v_/F_m_		Ф_PSII_		qP		NPQ	
	*F*	*P*	*F*	*P*	*F*	*P*	*F*	*P*	*F*	*P*	*F*	*P*	*F*	*P*	*F*	*P*	*F*	*P*	*F*	*P*
*H flabellatum*																				
*N*	147.181	<0.001	66.260	<0.001	72.059	<0.001	6.648	0.002	27.273	<0.001	0.998	2.061	0.136	52.224	<0.001	25.145	<0.001	4.175	0.020	0.998
*P*	20.419	<0.001	38.703	<0.001	3.634	0.032	0.774	0.465	8.677	<0.001	0.322	0.994	0.376	15.961	<0.001	4.609	0.014	1.902	0.158	0.322
(*N*×*P*)_1_	1.888	0.160	0.869	0.425	3.517	0.036	1.020	0.367	5.133	0.009	0.103	1.814	0.172	4.676	0.013	2.407	0.098	2.438	0.096	0.103
(*N*×*P*)_2_	0.151	0.860	2.570	0.085	3.462	0.038	0.950	0.392	2.804	0.068	0.452	1.206	0.306	3.182	0.048	0.579	0.563	0.482	0.620	0.452
*S*	22.062	<0.001	23.102	<0.001	0.930	0.400	0.636	0.533	1.270	0.288	0.601	0.331	0.720	44.438	<0.001	20.432	<0.001	1.041	0.359	0.601
(*N*×*S*)_1_	8.864	<0.001	3.909	0.025	0.679	0.511	0.680	0.510	0.614	0.545	0.615	0.929	0.400	0.868	0.425	0.375	0.689	0.271	0.763	0.615
(*N*×*S*)_2_	10.576	<0.001	5.794	0.005	0.196	0.823	0.777	0.464	2.469	0.093	0.609	1.753	0.182	0.324	0.725	0.997	0.375	0.094	0.910	0.609
(*P*×*S*)_1_	0.493	0.613	0.198	0.821	0.765	0.470	3.051	0.054	1.535	0.224	0.016	0.975	0.383	1.540	0.223	0.763	0.470	0.039	0.962	0.016
(*P*×*S*)_2_	0.513	0.601	1.468	0.238	0.777	0.464	0.119	0.888	0.469	0.628	0.484	1.570	0.216	0.393	0.677	3.320	0.043	0.313	0.733	0.484
*P*. *assamica*																				
*N*	134.277	<0.001	78.862	<0.001	44.227	<0.001	13.882	<0.001	16.856	<0.001	134.277	<0.001	78.862	<0.001	44.227	<0.001	13.882	<0.001	16.856	<0.001
*P*	37.592	<0.001	43.349	<0.001	2.369	0.102	1.546	0.221	3.427	0.039	37.592	<0.001	43.349	<0.001	2.369	0.102	1.546	0.221	3.427	0.039
(*N*×*P*)_1_	5.525	0.006	1.201	0.308	1.822	0.170	0.350	0.706	1.304	0.279	5.525	0.006	1.201	0.308	1.822	0.170	0.350	0.706	1.304	0.279
(*N*×*P*)_2_	4.127	0.021	2.374	0.102	0.060	0.942	1.140	0.326	0.409	0.666	4.127	0.021	2.374	0.102	0.060	0.942	1.140	0.326	0.409	0.666
*S*	3.886	0.026	27.937	<0.001	1.030	0.363	0.157	0.855	0.560	0.574	3.886	0.026	27.937	<0.001	1.030	0.363	0.157	0.855	0.560	0.574
(*N*×*S*)_1_	0.348	0.707	3.936	0.025	0.485	0.618	0.587	0.559	0.638	0.532	0.348	0.707	3.936	0.025	0.485	0.618	0.587	0.559	0.638	0.532
(*N*×*S*)_2_	0.118	0.889	6.267	0.003	0.335	0.717	0.262	0.771	0.633	0.535	0.118	0.889	6.267	0.003	0.335	0.717	0.262	0.771	0.633	0.535
(*P*×*S*)_1_	0.793	0.457	0.639	0.531	0.112	0.894	0.907	0.409	0.484	0.619	0.793	0.457	0.639	0.531	0.112	0.894	0.907	0.409	0.484	0.619
(*P*×*S*)_2_	0.009	0.991	1.065	0.351	1.062	0.352	0.066	0.936	0.350	0.706	0.009	0.991	1.065	0.351	1.062	0.352	0.066	0.936	0.350	0.706

EC, electrical conductivity; Chl, total chlorophyll concentration; N/P, N:P ratio; F_v_/F_m_, maximal photochemical efficiency of PSII; Ф_PSII_, actual photochemical efficiency of PSII; qP, photochemical quenching; NPQ, non-photochemical quenching; [N], [P] and [S] representing N, P and S concentrations, respectively.

### Chlorophyll concentrations

The two epiphytic bryophytes demonstrated similar trends between Chl concentration and fertilizer additions. Chl increased with increasing application levels and peaked at approximately 20 kg N ha^−1^ yr^−1^ (Fig 1D). While a decline in Chl with increasing P and S levels was distinct (Fig 1E and 1F). Particularly, there was an additive interaction for Chl between N and S under low fertilization levels (*N*×*S*, Table 1; *P*<0.05); P addition combined with N or S had limited effects on Chl (Table 1; *P*<0.05).

### Nutrient stoichiometry

**Fig 2:**
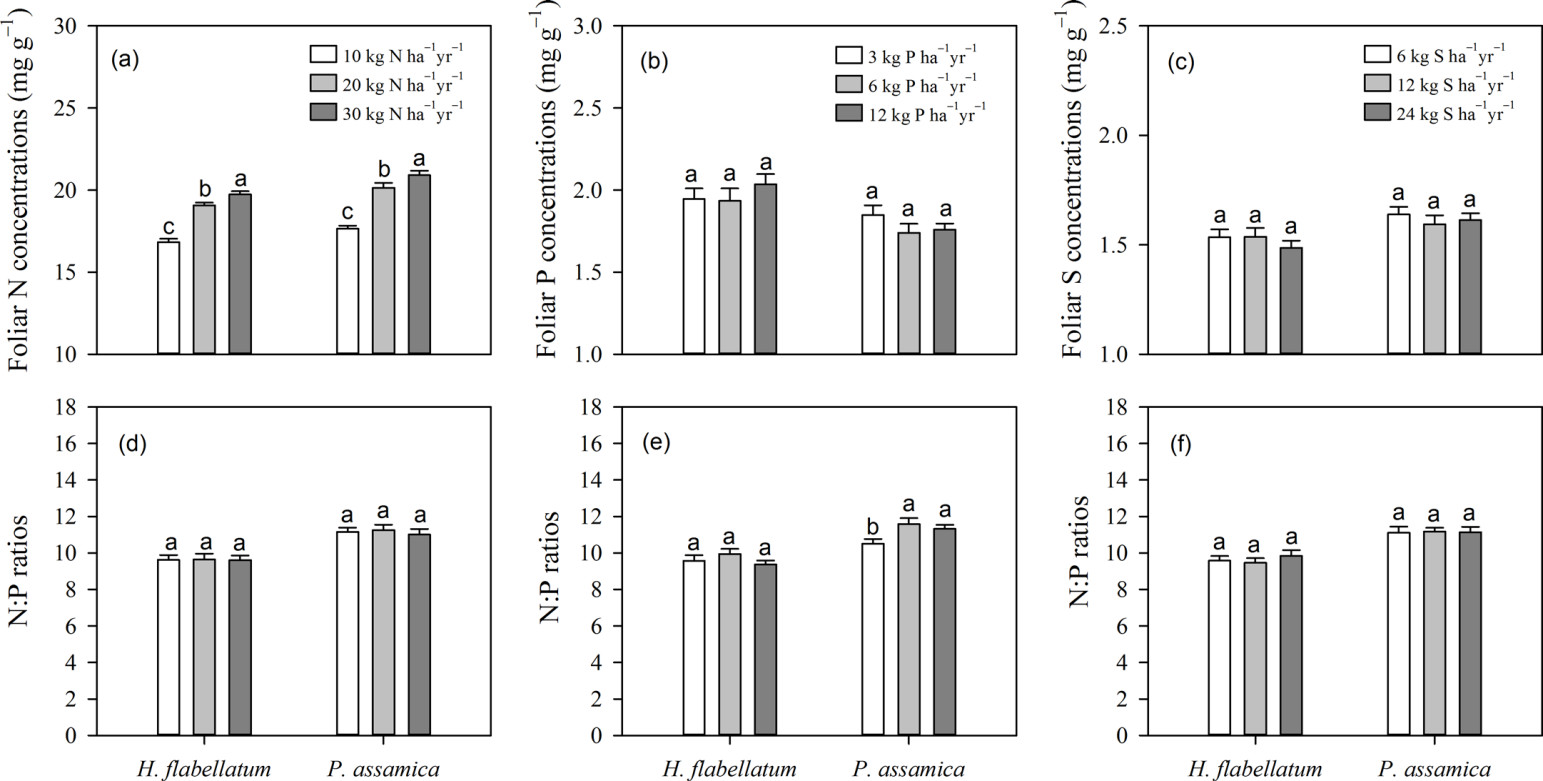
Multiple comparisons of different fertilizers and application levels on nutrient stoichiometry of *H*. *flabellatum* and *P*. *assamica* (means±se, *n* = 3). Three application levels are shown as in Fig 1. Letters above columns indicate significant differences (*P*≤0.05).

Capitula N concentration increased rapidly with increasing N addition at low-level addition, but the increment was less at higher application levels. N tended to be saturated at deposition rates over 30 kg N ha^−1^ yr^−1^ (Fig 2A). There was no significant difference in foliar P and S concentration at respective application levels in the two species (Fig 2B and 2C). However, P addition facilitated N uptake in *H*. *flabellatum* (*N*×*P*, Table 1; *P*<0.05). The two species coincidently displayed the lowest tissue P concentration at moderate levels of P addition. *H*. *flabellatum* had the highest tissue P concentration at the highest addition level, while foliar P concentration in *P*. *assamica* exhibited the opposite trend to P addition (Fig 2B). The relationship between foliar S concentrations and S addition levels was species specific, with the maximal and minimal concentrations of *H*. *flabellatum* and *P*. *assamica* reached at moderate addition level, respectively (Fig 2C). In addition, the N:P ratios of the two epiphytic bryophytes were not responsive to experimental nutrient gradients (Fig 2D–2F, except to P addition in *P*. *assamica*, *P* = 0.028).

### Chlorophyll *a* fluorescence signals

**Fig 3:**
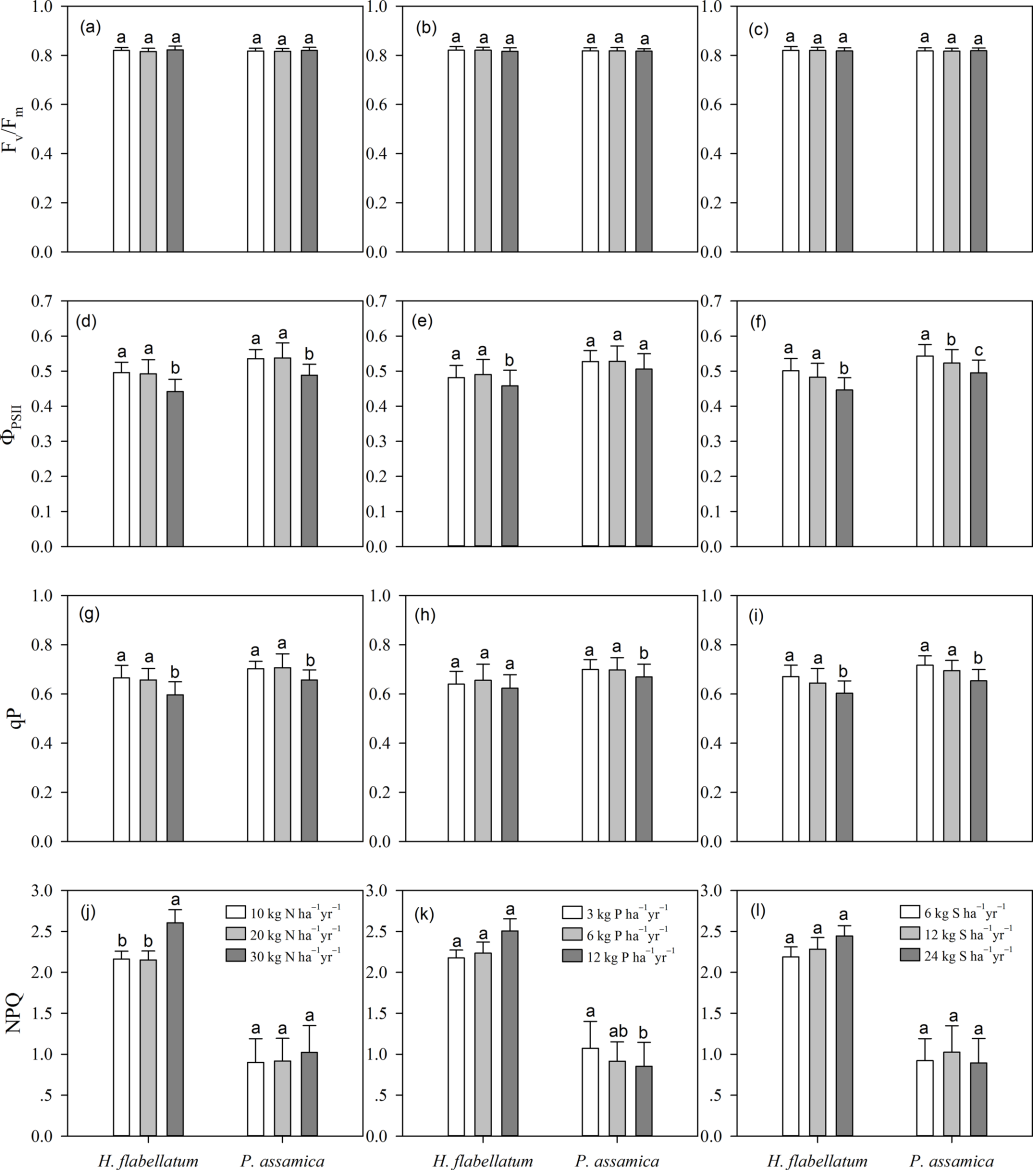
Multiple comparisons of different fertilizers and application levels on fluorescence signals of *H*. *flabellatum* and *P*. *assamica* (means±se, *n* = 3). Three application levels are shown as in Fig 1. Letters above columns indicate significant differences (*P*≤0.05).

F_v_/F_m_ did not differ significantly among fertilization treatments in the two species (Fig 3A–3C; Table 1; *P*>0.05). Φ_PSII_ and qP in the two species decreased abruptly at the highest N and S application levels (Fig 3D, 3F, 3G and 3I). Both parameters peaked at moderate levels of P addition. There was an additive interaction for Φ_PSII_ between N and P addition (Table 1; *P*<0.05). On the whole, NPQ increased with nutrient addition in *H*. *flabellatum*. By contrast, NPQ increased with N addition and decreased with P addition in *P*. *assamica*, and its highest value appeared at moderate level of S addition (Fig 3J–3L).

## Discussion

### Effects of simulated atmospheric depositions

Increased nutrient treatments triggered significant physiological responses in bryophytes. As reported in previous research [6], N toxicity leads to loss of membrane integrity and increases solute leakage. Fig 1D illustrated that a proper N loading (10–20 kg ha^−1^ yr^−1^) stimulates the formation of Chl in bryophytes because most foliar N is invested in photosynthetic apparatus. While excessive N uptake may degrade Chl and hinder photosynthesis, which is in agreement with previous reports that the optimal N influx for Chl formation in *Sphagnum magellanicum* appears at approximately 23 kg ha^−1^ yr^−1^ [32]. In addition, tissue N concentration does not increase linearly, and the retention capacity of N decreases with high N inputs (>30 kg ha^−1^ yr^−1^), which has been proven by earlier research in *Racomitrium lanuginosum* [6]. The saturating trend at high N input probably is a consequence of nutritional constraints caused by other nutrients, such as P and K [5]. F_v_/F_m_ is usually used as a proxy for stress. However, increased nutrient additions did not cause pronounced changes in F_v_/F_m_ (Fig 3A–3C; Table 1). These results suggest that fertilization treatments had a limited effect on PSII. In contrast, other authors have claimed that F_v_/F_m_ is significantly affected by fertilization treatments [17].

The enhanced availability of P poses serious threats to the physiological performances of two target bryophytes. Therefore, an increase in P availability may make species originally adapted to P-limited conditions no longer beneficial [33]. For example, at least some specialized species show competitive advantages only under P-limited rather than N-limited conditions. Bryophytes have a high capacity to absorb P [34], and previous studies also proved that most species, particularly those adapted to P-deficient environments, have no ability to down-regulate P uptake [35]. Whether the mechanism is storage or inability to down-regulate uptake, the foliar accumulation of P observed across many species highlights the importance of P acquirement, and hints at a potential evolutionary significance.

In general, P-deficient plants increase the rate of P uptake but reduce the rate of N uptake, and vice versa [36]. A meta-analysis demonstrated that combined N and P enrichment produces positive synergistic responses in most environments [37], which indicates that the supply and demand between N and P are in close balance under most conditions [14, 38, 39]. Unfortunately, data is still scarce on how the availability of one resource affects the supply of and demand for another [40]. Limited publications [8] provide insight into these interactions, suggesting that enhanced N supply increases phosphatase activity in bryophytes, which in turn enhances the availability of N [25]. Our study showed that N addition significantly affected the foliar N, P and S concentrations in two bryophytes (Table 1; *P*<0.05), while foliar P concentration was not affected profoundly by P or S addition (Table 1; *P*>0.1). Bryophytes accumulate nutrients directly through the influx of atmospheric deposition into living cells. Conversely, vascular epiphytes demonstrate a different pattern of nutrients uptake. For example, N uptake in *Tillandsia landbeckii* depends more on its availability (mainly from fog), whereas P uptake is primarily regulated by internal demand [41]. Therefore, considerable efforts are urgently needed to further study the mechanism of P supply and demand within epiphytic bryophytes.

S addition only had limited impacts on foliar S concentration in our case (Table 1; *P*>0.1). In contrast, high S treatment (20 kg ha^−1^ yr^−1^) increases the total S concentration by 70–80% in the uppermost 5 cm of the peat profile [42]. Moreover, S fertilization markedly decreased Φ_PSII_ and qP in the two bryophytes (Fig 3F and 3I), which may imply that high S treatment results in an accumulation of non-functional PSII centers. This is consistent with previous research that high loads of SO_4_^2−^ damage PSII in *Sphagnum balticum* [43]. However, SO_4_^2−^ appears to be harmless for photosynthesis in *Sphagnum recurvum*. [44]. Interestingly, the adverse effects of high S deposition may be relaxed or completely disappear when S is combined with N [43]. A plausible explanation is that high availability of N facilitates S storage or reduces its uptake.

### Potential limitation of ecological stoichiometry for indicating atmospheric depositions

Changes in foliar chemistry, particularly N concentration, are considered to be the most sensitive predictor of atmospheric N deposition [45]. Bryophytes generally show luxury consumption [46] due to their limited ability to regulate N uptake [6]. As a consequence, a positive correlation between tissue N concentration and increasing N supply has been extensively documented in a large number of species [6, 47] as well as in our case (Table 1; *P*<0.01).

However, sometimes the relationship between atmospheric N deposition and the N concentration in bryophytes is not so distinct [48], or has been shown to be species specific [4, 17]. In fact, bryophytes tend to become less efficient at sequestering N at high deposition levels [49], which may indicate tissue N saturation [50]. As such, our results support the view that only when N deposition increases to high levels will N exert devastating effects on bryophytes [50]. Similarly, specimens originating from high N deposition areas take up less N than those originating from low N deposition regions [51]. Therefore, decreased N uptake may be a long-term adaptation for bryophytes subjected to high N supply [51, 52]. Several authors have suggested that specimens subjected to long-term N enrichment display a plastic response to new environmental scenarios through higher tissue N concentration [53], or reduced N uptake [52] or physiological acclimation [51]. In accord with these explanations, bryophytes from areas with high background levels cope better with N enrichment than those from areas with low background levels [27], which together stresses the importance of considering the history of background N deposition when performing and interpreting N addition experiments. With respect to P and S, fertilization posed limited effects on their respective foliar concentrations (Table 1; *P*>0.1). In contrast, N addition facilitated the storage or uptake of P and S (Table 1; *P*<0.01). Therefore, it is too early to draw a unanimous conclusion concerning the saturation uptake and nutrient interaction in bryophytes.

### Feasibility of using N:P ratio to indicate nutrient limitation in epiphytic bryophytes

Ecological stoichiometry theory provides an integrative approach for the analysis of nutrient balance at different levels. For example, foliar nutrient concentrations and N:P ratios are widely used to assess nutrient status and potential nutrient-limitation [39], with relative ratios above 16 indicating P limitation and those below 14 indicating N limitation [54]. But the thresholds (i.e. critical values) derived from wetland communities are not applicable in other ecosystems [55], because thresholds vary considerably across plant groups [39, 56]. For example, the critical N:P value is beyond the confines of 15 and 30 in *Sphagnum* to indicate N or P limitation, respectively [5, 7]. More recently, Güsewell [39] proposed a broader range of N:P ratios (10–20) to indicate co-limitation of N and P in a community.

The overall N:P ratio of China’s flora (14.4±0.40, mean±se) is relatively higher than the global average (11.8±0.32) [56]. Foliar N concentrations in our study were 19.43±0.45 mg g^−1^, equaling to the maximal N concentration in a previous study [50]. But by comparing the N:P ratio (11.18±0.51) in our study with those from central-west and northern Europe [5, 47] and South America [23], we found that the discrepancy of low N:P ratio is mainly caused by relatively high P concentrations (1.81±0.11) rather than by low N concentrations. Possible explanations for the lower N:P ratios are that this region has suffered from disproportionate input of N and P, or that the two studied epiphytic bryophytes may be still N-limited despite high capitula N concentrations [57]. Therefore, it is more reasonable to use the N:P ratio, rather than the absolute concentration, to indicate the type of nutrient limitation in epiphytic bryophytes.

It is not possible to conclude with confidence that local epiphytic bryoflora are P-limited despite local soil status of P-deficiency [28]. We postulated that atmospheric P supply from related industries may pose important impacts on the nutrient stoichiometry of local epiphytic bryoflora, because the input of atmospheric P deposition from distant regions plays an important role in P-deficient forest ecosystems [58]. Interestingly, mining of phosphorus compounds for fertilizer and related industries has boomed extraordinarily in Yunnan province in recent decades [59].

Several authors [5] have hypothesized that N:P ratios in autotrophs should closely reflect N and P supplies. In the study region, although the main hosts (*C*. *wattii*, *L*. *xylocarpus* and *Schima noronhae*) demonstrated variation in foliar N:P ratios, they still exhibited a high degree of ‘stoichiometric homeostasis’ (data not shown, y = 18.91x^0.66^, *P*<0.001, where y and x represent foliar N and P concentrations, respectively). But in some cases, the correspondence between biomass N:P ratio and the relative availability of N and P does not match faithfully due to homeostatic regulation or nutrient recycling [25, 60]. For example, N:P ratio in hummock and lawn *Sphagnum* species increased steeply at low atmospheric N input, whereas the trend became gradual above a depositional threshold of approximately 10 kg ha^−1^ yr^−1^ as a consequence of N saturation [5]. Unlike the result of early studies [57], the N:P ratios of the two epiphytic bryophytes exhibited limited response to nutrient gradients (except the response to P addition in *P*. *assamica*, *P* = 0.028). Overall, the above phenomenon suggested that bryophytes and vascular plants may respond to nutrient availability differently. Our results confirmed that one should prudently apply the critical ratios developed from vascular plants to epiphytic bryophytes.

### Necessity of long-term simulated experiments with multiple atmospheric depositions

The outcomes of simulated fertilization experiments are time-dependent [25, 40]. So far, most N manipulative experiments have been performed over a relatively short-term scale but have highlighted the necessity of long-term measurements [24, 27] because the ‘build up’ impacts of low N addition may take years to be detectable [61]. Therefore, short-term studies may underestimate the negative effects of chronic and low-level N deposition [62]. Furthermore, some previous N fertilization experiments have not simulated realistic N deposition, as N has been employed only a few times with high concentrations during the growing season [6, 18], which may cause unrealistic responses compared with ‘real world’ N deposition that occurs year-round at high frequencies and low concentrations.

An approach to quantify the pollution deposition is to determine the critical load, and a critical load of 10 kg N ha^−1^ yr^−1^ has been proposed for Europe and high Arctic heath [5, 14]. By contrast, the critical load of N for montane summit ecosystems, based on effects on bryophytes and lichens, may be as low as 5–10 kg ha^−1^ yr^−1^ [3]. There are several sources of uncertainty in our assessment of empirical critical loads, including data gaps, time lags, effect of multiple stressors, etc. For example, it is difficult to determine the actual critical load of N deposition for forest ecosystems only on the basis of a short-term fertilization study owing to a long lag time in response to N treatments. If a response is observed over a relatively short period of time (i.e. years), it is almost certain that the threshold is below the total N input at the treatment site. As a consequence, it is impossible to further refine the threshold. In line with this, long-term experiments over decades suggest that thresholds for an explicit effect may be lower with increased duration of treatments [62], and there may be simply no threshold for these changes if the experimental duration lasts for long enough. Therefore, the cumulative effect of N addition should be included in calculating the critical load values in field experiments.

### Implications for N management strategies in montane forest

Since the late 1980s, rates of N deposition have leveled off in Europe with the implementation of stricter legislation to limit atmospheric pollution. In contrast, emissions of N pollution in China have been increasing with intensive agricultural and industrial activities [3, 63]. Overall, the empirical data from the present study indicate that the expert-based range of critical load of N deposition is set too loose for montane forest ecosystems. For example, the recommended critical load for montane grassland is 10–15 kg N ha^−1^ yr^−1^ [16]. However, Nordin and colleagues [64] observed that vegetation changes in key ecosystem components had occurred even at a lower rate of 6 kg N ha^−1^ yr^−1^, especially in regions with low background N deposition rates.

Our tentative results demonstrated that the critical load of N for the epiphytic bryophytes lies below 20 kg ha^−1^ yr^−1^, according to the guideline of detrimental impacts on EC, Chl, Φ_PSII_, and qP. Moreover, the response of bryophytes to fertilization is species specific [4, 14]. As a result, N deposition may cause species replacement within bryophyte communities by competitive exclusion. For example, in boreal forests, *Hylocomium splendens* starts to decline at an input rate of >10 kg N ha^−1^ yr^−1^ [65], whereas *Brachythecium* spp. and *Plagiothecium* spp., two nitrophilous genera, are abundant in nutrient-rich habitats after 47 years of N application [66]. Obviously, different sensitivity of bryophytes to elevated N deposition prevents generalization of the thresholds in different ecosystems. Therefore, the effect of N deposition on physiological responses needs further investigation for establishing a proper threshold of N management strategies in montane forest ecosystems.

### A guide for future investigations

Fertilization experiments are regarded as the best approach to identify the type of nutrient limitation, but they do not always result in consistent results. The outcomes depend on experimental methods, such as the duration, frequency and intensity of fertilization or the variables measured. For example, tissue N in bryophytes is more sensitive to simulated concentrations than to deposition doses [29, 67]. Cumulative dose is a recently developed approach [19], which may provide novel insights into how damage develops over time under elevated deposition rates, because it integrates time, input rate and ambient deposition [61]. Furthermore, the applied N form (e.g. dry and wet, reduced and oxidized) [20, 67] and environmental and climatic factors (altitude and latitude, altered precipitation, global warming and elevated concentrations of CO_2_) [56, 68] should also be taken into account in future investments, due to their importance in modifying the response of bryophytes to N enrichment. Particularly, data on the effects of S deposition are extremely scarce, and these studies should be emphasized due to interactive effects of S with N and P.

## Conclusions

Our field experiment suggested that enhanced atmospheric depositions imposed detrimental impacts on physiological performance of two epiphytic bryophytes, whereas the metabolic burden imposed by excessive N cannot be completely alleviated by P and S addition. In general, foliar N was not a robust indicator of N deposition in bryophytes concerning the saturation uptake and the species-specific response of N concentration to application regimes. Moreover, none of the species showed a pronounced relationship between N:P ratio and N and P addition, indicating that N:P ratio is not yet an ideal candidate for bioindication programs of atmospheric deposition. Our results confirmed that one can not assess the type of nutrient limitation simply by calculating the N:P ratio for bryophytes and applying the critical values developed from vascular plants to bryophytes. Nevertheless, further studies are necessary to better understand the underlying mechanisms leading to high levels of foliar P concentration in the subtropical epiphytic bryoflora that is traditionally regarded as P-deficiency.

## Supporting Information

S1 Table Design of orthogonal table *L*_27_(313) for three factors with three application levels each. (DOCX)

S2 Table Mean values (standard errors in parentheses, *n* = 3) of physiological parameters in *H*. *flabellatum* and *P*. *assamica* (*Continued* Table) to fertilizer additions. (DOCX)
