# Supplementary material for: Physiological Responses of Two Epiphytic Bryophytes to Nitrogen, Phosphorus and Sulfur Addition in a Subtropical Montane Cloud Forest
Source: PLoS One. 2016 Aug 25;11(8):e0161492. doi: 10.1371/journal.pone.0161492 (PMC4999294; doi:10.1371/journal.pone.0161492)
Supplement: S1 Table — (DOCX) [file pone.0161492.s001.docx]

S1 Table. Design of orthogonal table *L*_27_(_3_^13^) for three factors with three application levels each.

Factors

| Levels | *N* | *P* | (*N*×*P*)_1_^b^ | (*N*×*P*)_2_ | *S* | (*N*×*S*)_1_ | (*N*×*S*)_2_ | (*P*×*S*)_1_ | Blank column | Blank column | (*P*×*S*)_2_ | Treatments |
| --- | --- | --- | --- | --- | --- | --- | --- | --- | --- | --- | --- | --- |
| 1  No. | 1(10)^a^ | 1(3) | 1 | 1 | 1(6) | 1 | 1 | 1 | 1 | 1 | 1 | *N*_1_*P*_1_*S*_1_^c^ |
| 2 | 1 | 1 | 1 | 1 | 2(12) | 2 | 2 | 2 | 2 | 2 | 2 | *N*_1_*P*_1_*S*_2_ |
| 3 | 1 | 1 | 1 | 1 | 3(24) | 3 | 3 | 3 | 3 | 3 | 3 | *N*_1_*P*_1_*S*_3_ |
| 4 | 1 | 2(6) | 2 | 2 | 1 | 1 | 1 | 2 | 2 | 2 | 3 | *N*_1_*P*_2_*S*_1_ |
| 5 | 1 | 2 | 2 | 2 | 2 | 2 | 2 | 3 | 3 | 3 | 1 | *N*_1_*P*_2_*S*_2_ |
| 6 | 1 | 2 | 2 | 2 | 3 | 3 | 3 | 1 | 1 | 1 | 2 | *N*_1_*P*_2_*S*_3_ |
| 7 | 1 | 3(12) | 3 | 3 | 1 | 1 | 1 | 3 | 3 | 3 | 2 | *N*_1_*P*_3_*S*_1_ |
| 8 | 1 | 3 | 3 | 3 | 2 | 2 | 2 | 1 | 1 | 1 | 3 | *N*_1_*P*_3_*S*_2_ |
| 9 | 1 | 3 | 3 | 3 | 3 | 3 | 3 | 2 | 2 | 2 | 1 | *N*_1_*P*_3_*S*_3_ |
| 10 | 2(20) | 1 | 2 | 3 | 1 | 2 | 3 | 1 | 3 | 3 | 1 | *N*_2_*P*_1_*S*_1_ |
| 11 | 2 | 1 | 2 | 3 | 2 | 3 | 1 | 2 | 1 | 1 | 2 | *N*_2_*P*_1_*S*_2_ |
| 12 | 2 | 1 | 2 | 3 | 3 | 1 | 2 | 3 | 2 | 2 | 3 | *N*_2_*P*_1_*S*_3_ |
| 13 | 2 | 2 | 3 | 1 | 1 | 2 | 3 | 2 | 1 | 1 | 3 | *N*_2_*P*_2_*S*_1_ |
| 14 | 2 | 2 | 3 | 1 | 2 | 3 | 1 | 3 | 2 | 2 | 1 | *N*_2_*P*_2_*S*_2_ |
| 15 | 2 | 2 | 3 | 1 | 3 | 1 | 2 | 1 | 3 | 3 | 2 | *N*_2_*P*_2_*S*_3_ |
| 16 | 2 | 3 | 1 | 2 | 1 | 2 | 3 | 3 | 2 | 2 | 2 | *N*_2_*P*_3_*S*_1_ |
| 17 | 2 | 3 | 1 | 2 | 2 | 3 | 1 | 1 | 3 | 3 | 3 | *N*_2_*P*_3_*S*_2_ |
| 18 | 2 | 3 | 1 | 2 | 3 | 1 | 2 | 2 | 1 | 1 | 1 | *N*_2_*P*_3_*S*_3_ |
| 19 | 3(30) | 1 | 3 | 2 | 1 | 3 | 2 | 1 | 2 | 2 | 1 | *N*_3_*P*_1_*S*_1_ |
| 20 | 3 | 1 | 3 | 2 | 2 | 1 | 3 | 2 | 3 | 3 | 2 | *N*_3_*P*_1_*S*_2_ |
| 21 | 3 | 1 | 3 | 2 | 3 | 2 | 1 | 3 | 1 | 1 | 3 | *N*_3_*P*_1_*S*_3_ |
| 22 | 3 | 2 | 1 | 3 | 1 | 3 | 2 | 2 | 3 | 3 | 3 | *N*_3_*P*_2_*S*_1_ |
| 23 | 3 | 2 | 1 | 3 | 2 | 1 | 3 | 3 | 1 | 1 | 1 | *N*_3_*P*_2_*S*_2_ |
| 24 | 3 | 2 | 1 | 3 | 3 | 2 | 1 | 1 | 2 | 2 | 2 | *N*_3_*P*_2_*S*_3_ |
| 25 | 3 | 3 | 2 | 1 | 1 | 3 | 2 | 3 | 1 | 1 | 2 | *N*_3_*P*_3_*S*_1_ |
| 26 | 3 | 3 | 2 | 1 | 2 | 1 | 3 | 1 | 2 | 2 | 3 | *N*_3_*P*_3_*S*_2_ |
| 27 | 3 | 3 | 2 | 1 | 3 | 2 | 1 | 2 | 3 | 3 | 1 | *N*_3_*P*_3_*S*_3_ |

^a^ different experimental levels (practical application dose in parentheses, kg ha^−1^ yr^−1^); ^b^ interactions between factors; ^c^ treatments of three different factors with three levels each.
