# Supplementary material for: Physiological Responses of Two Epiphytic Bryophytes to Nitrogen, Phosphorus and Sulfur Addition in a Subtropical Montane Cloud Forest
Source: PLoS One. 2016 Aug 25;11(8):e0161492. doi: 10.1371/journal.pone.0161492 (PMC4999294; doi:10.1371/journal.pone.0161492)
Supplement: S2 Table — (DOCX) [file pone.0161492.s002.docx]

S2 Table. Mean values (standard errors in parentheses, *n*=3) of physiological parameters in *H. flabellatum* and *P. assamica* (Continued Table) to fertilizer additions.

| Treatments | EC | Chl | [N] | [P] | [S] | N/P | F_v_/F_m_ | Ф_PSII_ | qP | NPQ |
| --- | --- | --- | --- | --- | --- | --- | --- | --- | --- | --- |
| N_1_P_1_S_1_ | 7.39(0.81) | 4.03(0.10) | 17.02(0.28) | 2.09(0.13) | 1.39(0.06) | 8.19(0.42) | 0.82(0.01) | 0.51(0.02) | 0.70(0.03) | 2.18(0.53) |
| N_1_P_1_S_2_ | 7.41(0.66) | 3.82(0.11) | 15.63(1.16) | 1.78(0.12) | 1.20(0.09) | 8.76(0.23) | 0.82(0.02) | 0.49(0.01) | 0.63(0.03) | 1.73(0.07) |
| N_1_P_1_S_3_ | 8.32(0.46) | 3.70(0.13) | 16.54(0.52) | 1.77(0.24) | 1.28(0.01) | 9.66(1.09) | 0.82(0.01) | 0.47(0.01) | 0.61(0.05) | 1.97(0.50) |
| N_1_P_2_S_1_ | 7.65(0.91) | 3.87(0.11) | 16.79(0.81) | 1.69(0.06) | 1.48(0.06) | 9.95(0.31) | 0.82(0.01) | 0.52(0.02) | 0.70(0.02) | 2.17(0.55) |
| N_1_P_2_S_2_ | 7.69(0.47) | 3.72(0.16) | 17.13(0.38) | 1.57(0.16) | 1.42(0.04) | 11.10(0.83) | 0.82(0.01) | 0.52(0.01) | 0.72(0.06) | 2.79(0.57) |
| N_1_P_2_S_3_ | 10.05(0.75) | 3.57(0.10) | 17.14(0.43) | 1.65(0.14) | 1.40(0.09) | 10.53(1.00) | 0.81(0.01) | 0.48(0.02) | 0.65(0.04) | 2.26(0.51) |
| N_1_P_3_S_1_ | 8.91(0.28) | 3.40(0.19) | 16.76(0.95) | 1.66(0.21) | 1.45(0.09) | 10.29(0.85) | 0.83(0.02) | 0.52(0.03) | 0.67(0.05) | 1.95(0.26) |
| N_1_P_3_S_2_ | 9.75(0.24) | 3.57(0.11) | 17.90(0.12) | 2.13(0.12) | 1.46(0.06) | 8.46(0.51) | 0.82(0.00) | 0.52(0.01) | 0.69(0.01) | 1.78(0.33) |
| N_1_P_3_S_3_ | 10.40(0.47) | 3.25(0.18) | 16.59(0.58) | 1.73(0.13) | 1.27(0.03) | 9.66(0.47) | 0.82(0.01) | 0.44(0.02) | 0.61(0.04) | 2.62(0.40) |
| N_2_P_1_S_1_ | 7.43(0.57) | 4.81(0.13) | 19.98(0.22) | 1.98(0.10) | 1.61(0.08) | 10.12(0.47) | 0.81(0.01) | 0.53(0.03) | 0.70(0.02) | 1.89(0.04) |
| N_2_P_1_S_2_ | 8.23(0.78) | 4.60(0.11) | 19.35(0.36) | 2.25(0.13) | 1.55(0.10) | 8.64(0.36) | 0.83(0.00) | 0.51(0.03) | 0.66(0.04) | 1.96(0.36) |
| N_2_P_1_S_3_ | 10.07(0.52) | 4.56(0.20) | 18.75(0.57) | 1.72(0.29) | 1.57(0.03) | 11.46(1.75) | 0.81(0.01) | 0.48(0.02) | 0.66(0.05) | 2.64(0.20) |
| N_2_P_2_S_1_ | 9.10(0.98) | 4.50(0.08) | 19.04(0.54) | 1.99(0.20) | 1.49(0.06) | 9.78(1.03) | 0.83(0.01) | 0.53(0.02) | 0.69(0.01) | 1.67(0.60) |
| N_2_P_2_S_2_ | 8.74(1.07) | 4.51(0.12) | 19.24(0.76) | 1.79(0.23) | 1.66(0.08) | 10.96(0.95) | 0.82(0.01) | 0.52(0.02) | 0.70(0.05) | 2.07(0.51) |
| N_2_P_2_S_3_ | 7.96(0.38) | 4.24(0.09) | 18.62(0.51) | 2.25(0.11) | 1.56(0.09) | 8.35(0.63) | 0.82(0.01) | 0.48(0.03) | 0.63(0.03) | 1.92(0.34) |
| N_2_P_3_S_1_ | 11.23(0.57) | 4.29(0.11) | 18.36(0.36) | 1.86(0.09) | 1.47(0.03) | 9.89(0.40) | 0.81(0.01) | 0.50(0.02) | 0.67(0.03) | 2.19(0.48) |
| N_2_P_3_S_2_ | 11.71(0.60) | 4.16(0.08) | 19.67(0.55) | 2.21(0.18) | 1.70(0.11) | 8.98(0.56) | 0.81(0.02) | 0.45(0.01) | 0.63(0.04) | 2.44(0.11) |
| N_2_P_3_S_3_ | 10.54(0.75) | 4.07(0.15) | 18.61(0.25) | 2.22(0.25) | 1.48(0.07) | 8.57(0.92) | 0.80(0.01) | 0.42(0.01) | 0.59(0.04) | 2.57(1.31) |
| N_3_P_1_S_1_ | 13.01(0.94) | 4.79(0.23) | 18.62(0.42) | 2.01(0.27) | 1.40(0.07) | 9.57(1.23) | 0.82(0.03) | 0.47(0.03) | 0.65(0.03) | 2.43(0.60) |
| N_3_P_1_S_2_ | 11.75(0.59) | 4.37(0.07) | 18.94(0.25) | 2.12(0.26) | 1.46(0.12) | 9.19(0.98) | 0.83(0.02) | 0.45(0.00) | 0.58(0.05) | 2.60(0.61) |
| N_3_P_1_S_3_ | 15.46(0.43) | 3.81(0.08) | 18.55(0.14) | 1.78(0.10) | 1.45(0.01) | 10.49(0.48) | 0.83(0.02) | 0.43(0.02) | 0.58(0.03) | 2.18(0.71) |
| N_3_P_2_S_1_ | 11.88(0.62) | 4.42(0.09) | 20.33(0.92) | 2.29(0.47) | 1.73(0.19) | 9.46(1.45) | 0.82(0.02) | 0.49(0.02) | 0.67(0.07) | 2.33(0.48) |
| N_3_P_2_S_2_ | 14.63(1.08) | 4.21(0.11) | 20.33(0.35) | 2.12(0.16) | 1.68(0.07) | 9.68(0.74) | 0.83(0.01) | 0.47(0.03) | 0.60(0.03) | 1.99(1.57) |
| N_3_P_2_S_3_ | 18.68(0.62) | 3.63(0.08) | 19.79(0.11) | 2.05(0.05) | 1.68(0.04) | 9.65(0.20) | 0.83(0.00) | 0.40(0.03) | 0.54(0.04) | 2.91(0.50) |
| N_3_P_3_S_1_ | 12.96(0.32) | 4.09(0.10) | 20.75(0.16) | 2.33(0.11) | 1.80(0.01) | 8.93(0.40) | 0.82(0.01) | 0.44(0.02) | 0.59(0.06) | 2.88(1.40) |
| N_3_P_3_S_2_ | 15.12(1.75) | 3.16(0.24) | 19.77(0.38) | 2.11(0.06) | 1.71(0.10) | 9.37(0.27) | 0.81(0.01) | 0.42(0.02) | 0.59(0.02) | 3.19(0.64) |
| N_3_P_3_S_3_ | 21.01(0.89) | 3.34(0.19) | 20.55(0.17) | 2.05(0.20) | 1.70(0.03) | 10.17(0.85) | 0.82(0.01) | 0.41(0.02) | 0.57(0.05) | 2.93(0.62) |

S2 Table (*Continued*).

| Treatments | EC | Chl | [N] | [P] | [S] | N/P | F_v_/F_m_ | Ф_PSII_ | qP | NPQ |
| --- | --- | --- | --- | --- | --- | --- | --- | --- | --- | --- |
| N_1_P_1_S_1_ | 4.32(0.22) | 4.89(0.14) | 17.67(0.27) | 1.58(0.03) | 1.44(0.05) | 11.19(0.34) | 0.80(0.01) | 0.54(0.02) | 0.73(0.02) | 0.85(0.21) |
| N_1_P_1_S_2_ | 4.52(0.31) | 4.58(0.17) | 17.24(0.66) | 1.59(0.09) | 1.36(0.14) | 10.90(0.83) | 0.82(0.01) | 0.52(0.01) | 0.70(0.02) | 1.12(0.27) |
| N_1_P_1_S_3_ | 4.70(0.19) | 4.39(0.21) | 17.45(0.83) | 1.65(0.04) | 1.50(0.10) | 10.55(0.33) | 0.81(0.01) | 0.52(0.01) | 0.68(0.01) | 1.20(0.54) |
| N_1_P_2_S_1_ | 4.46(0.36) | 4.68(0.18) | 17.27(0.70) | 1.58(0.02) | 1.45(0.07) | 10.90(0.38) | 0.83(0.00) | 0.56(0.02) | 0.72(0.03) | 0.72(0.11) |
| N_1_P_2_S_2_ | 5.01(0.11) | 4.47(0.23) | 17.60(0.60) | 1.60(0.14) | 1.42(0.06) | 11.11(0.69) | 0.82(0.01) | 0.54(0.03) | 0.70(0.02) | 1.04(0.27) |
| N_1_P_2_S_3_ | 5.12(0.14) | 4.12(0.21) | 17.84(0.66) | 1.46(0.15) | 1.54(0.11) | 12.45(1.09) | 0.81(0.02) | 0.51(0.02) | 0.68(0.03) | 0.80(0.26) |
| N_1_P_3_S_1_ | 5.51(0.21) | 3.94(0.24) | 18.35(0.61) | 1.76(0.05) | 1.59(0.08) | 10.47(0.57) | 0.82(0.01) | 0.57(0.02) | 0.74(0.00) | 0.83(0.23) |
| N_1_P_3_S_2_ | 5.47(0.12) | 4.18(0.20) | 18.16(0.58) | 1.70(0.08) | 1.48(0.05) | 10.68(0.18) | 0.83(0.02) | 0.55(0.02) | 0.70(0.03) | 0.77(0.21) |
| N_1_P_3_S_3_ | 5.79(0.14) | 3.71(0.22) | 17.22(0.81) | 1.45(0.14) | 1.52(0.08) | 12.09(1.28) | 0.82(0.01) | 0.51(0.02) | 0.67(0.04) | 0.78(0.27) |
| N_2_P_1_S_1_ | 4.56(0.28) | 5.99(0.10) | 20.09(1.07) | 2.01(0.19) | 1.60(0.07) | 10.23(1.29) | 0.83(0.01) | 0.58(0.02) | 0.76(0.01) | 1.21(0.29) |
| N_2_P_1_S_2_ | 4.99(0.40) | 5.81(0.16) | 18.95(0.80) | 1.85(0.04) | 1.55(0.09) | 10.25(0.65) | 0.81(0.01) | 0.56(0.02) | 0.74(0.04) | 0.86(0.29) |
| N_2_P_1_S_3_ | 5.31(0.53) | 5.64(0.26) | 19.06(0.70) | 1.79(0.06) | 1.56(0.10) | 10.65(0.54) | 0.82(0.01) | 0.53(0.01) | 0.67(0.01) | 0.73(0.07) |
| N_2_P_2_S_1_ | 5.05(0.49) | 5.68(0.09) | 20.01(1.18) | 1.62(0.22) | 1.69(0.17) | 12.84(1.87) | 0.81(0.01) | 0.57(0.03) | 0.75(0.02) | 0.85(0.18) |
| N_2_P_2_S_2_ | 5.01(0.55) | 5.61(0.14) | 21.12(1.02) | 1.77(0.05) | 1.72(0.09) | 11.93(0.71) | 0.82(0.01) | 0.57(0.02) | 0.75(0.02) | 0.99(0.43) |
| N_2_P_2_S_3_ | 5.36(0.50) | 5.26(0.14) | 19.86(0.38) | 1.83(0.11) | 1.60(0.02) | 10.91(0.48) | 0.82(0.01) | 0.54(0.03) | 0.71(0.04) | 1.02(0.22) |
| N_2_P_3_S_1_ | 6.00(0.25) | 5.32(0.17) | 20.48(0.92) | 1.90(0.06) | 1.72(0.05) | 10.78(0.31) | 0.81(0.00) | 0.54(0.03) | 0.71(0.04) | 0.85(0.16) |
| N_2_P_3_S_2_ | 5.98(0.26) | 5.09(0.08) | 20.57(1.04) | 1.76(0.02) | 1.60(0.07) | 11.69(0.74) | 0.81(0.01) | 0.49(0.03) | 0.66(0.05) | 0.91(0.37) |
| N_2_P_3_S_3_ | 6.24(0.64) | 4.95(0.24) | 21.11(1.06) | 1.77(0.13) | 1.78(0.08) | 11.95(0.40) | 0.82(0.01) | 0.46(0.03) | 0.62(0.06) | 0.86(0.39) |
| N_3_P_1_S_1_ | 6.46(0.69) | 5.93(0.18) | 20.86(0.51) | 2.03(0.07) | 1.71(0.07) | 10.32(0.42) | 0.83(0.01) | 0.52(0.01) | 0.69(0.01) | 1.13(0.26) |
| N_3_P_1_S_2_ | 7.06(0.30) | 5.44(0.17) | 20.90(0.78) | 1.97(0.13) | 1.61(0.07) | 10.65(0.48) | 0.82(0.02) | 0.51(0.02) | 0.69(0.01) | 1.54(0.09) |
| N_3_P_1_S_3_ | 7.62(0.49) | 4.57(0.14) | 19.77(0.38) | 2.15(0.41) | 1.63(0.09) | 9.86(1.69) | 0.82(0.01) | 0.48(0.03) | 0.64(0.03) | 1.01(0.21) |
| N_3_P_2_S_1_ | 7.23(0.46) | 5.53(0.14) | 21.77(1.09) | 1.92(0.33) | 1.82(0.15) | 11.99(1.96) | 0.82(0.02) | 0.52(0.02) | 0.69(0.02) | 1.11(0.24) |
| N_3_P_2_S_2_ | 7.41(0.51) | 5.18(0.17) | 22.38(1.27) | 2.01(0.07) | 1.88(0.17) | 11.14(0.54) | 0.81(0.01) | 0.49(0.03) | 0.68(0.06) | 0.92(0.12) |
| N_3_P_2_S_3_ | 7.37(0.67) | 4.30(0.14) | 20.46(0.55) | 1.87(0.01) | 1.73(0.02) | 10.92(0.31) | 0.83(0.00) | 0.45(0.03) | 0.60(0.03) | 0.80(0.15) |
| N_3_P_3_S_1_ | 9.47(0.68) | 5.02(0.15) | 20.19(0.39) | 1.83(0.14) | 1.73(0.03) | 11.19(0.92) | 0.83(0.01) | 0.50(0.02) | 0.65(0.03) | 0.76(0.42) |
| N_3_P_3_S_2_ | 9.34(0.50) | 3.61(0.33) | 21.16(0.82) | 1.75(0.13) | 1.72(0.08) | 12.16(0.45) | 0.81(0.01) | 0.47(0.04) | 0.65(0.02) | 1.08(0.39) |
| N_3_P_3_S_3_ | 10.57(0.92) | 3.84(0.24) | 20.74(0.68) | 1.91(0.07) | 1.66(0.14) | 10.87(0.58) | 0.81(0.01) | 0.46(0.03) | 0.62(0.03) | 0.85(0.42) |

Abbreviations as in Table 1.
